# Analysis and applications of respiratory surface EMG: report of a round table meeting

**DOI:** 10.1186/s13054-023-04779-x

**Published:** 2024-01-02

**Authors:** A. H. Jonkman, R. S. P. Warnaar, W. Baccinelli, N. M. Carbon, R. F. D’Cruz, J. Doorduin, J. L. M. van Doorn, J. Elshof, L. Estrada-Petrocelli, J. Graßhoff, L. M. A. Heunks, A. A. Koopman, D. Langer, C. M. Moore, J. M. Nunez Silveira, E. Petersen, D. Poddighe, M. Ramsay, A. Rodrigues, L. H. Roesthuis, A. Rossel, A. Torres, M. L. Duiverman, E. Oppersma

**Affiliations:** 1 Department of Intensive Care Medicine, Erasmus Medical Center, Rotterdam, The Netherlands; 2 Cardiovascular and Respiratory Physiology, TechMed Centre, University of Twente, Enschede, The Netherlands; 3 Netherlands eScience Center, Amsterdam, The Netherlands; 4 Department of Anesthesiology, Friedrich Alexander-Universität Erlangen-Nürnberg, Uniklinikum Erlangen, Erlangen, Germany; 5 Lane Fox Clinical Respiratory Physiology Research Centre, Guy’s and St Thomas’ NHS Foundation Trust, London, UK; 6 Department of Neurology, Donders Institute for Brain, Cognition and Behaviour, Radboud University Medical Center, Nijmegen, The Netherlands; 7 Department of Pulmonary Diseases/Home Mechanical Ventilation, University of Groningen, University Medical Center Groningen, Groningen, The Netherlands; 8 Facultad de Ingeniería and Secretaría Nacional de Ciencia, Tecnología e Innovación (SENACYT) - Sistema Nacional de Investigación (SNI), Universidad Latina de Panamá (ULATINA), Panama, Panama; 9 Fraunhofer Research Institution for Individualized and Cell-Based Medical Engineering, Lübeck, Germany; 10 Department of Intensive Care, Radboud University Medical Center, Nijmegen, The Netherlands; 11 Division of Paediatric Critical Care Medicine, Department of Paediatrics, Beatrix Children’s Hospital, University Medical Center Groningen, Groningen, The Netherlands; 12 Research Group for Rehabilitation in Internal Disorders, Department of Rehabilitation Sciences, KU Leuven, 3000 Leuven, Belgium; 13 Hospital Italiano de Buenos Aires, Unidad de Terapia Intensiva, Ciudad de Buenos Aires, Argentina; 14 Technical University of Denmark (DTU), DTU Compute, 2800 Kgs. Lyngby, Denmark; 15 Keenan Centre for Biomedical Research, Li Ka Shing Knowledge Institute, Unity Health Toronto, Toronto, ON Canada; 16 Department of Acute Medicine, Geneva University Hospitals, Geneva, Switzerland; 17 Institut de Bioenginyeria de Catalunya (IBEC), Barcelona Institute of Science and Technology (BIST) and Biomedical Research Networking Centre in Bioengineering, Biomaterials and Nanomedicine (CIBER-BBN), Universitat Politècnica de Catalunya BarcelonaTech (UPC), Barcelona, Spain

## Abstract

Surface electromyography (sEMG) can be used to measure the electrical activity of the respiratory muscles. The possible applications of sEMG span from patients suffering from acute respiratory failure to patients receiving chronic home mechanical ventilation, to evaluate muscle function, titrate ventilatory support and guide treatment. However, sEMG is mainly used as a monitoring tool for research and its use in clinical practice is still limited—in part due to a lack of standardization and transparent reporting. During this round table meeting, recommendations on data acquisition, processing, interpretation, and potential clinical applications of respiratory sEMG were discussed. This paper informs the clinical researcher interested in respiratory muscle monitoring about the current state of the art on sEMG, knowledge gaps and potential future applications for patients with respiratory failure.

**Supplementary Information:**

The online version contains supplementary material available at 10.1186/s13054-023-04779-x.

## Introduction

Respiratory electromyography (EMG) has been used in intensive care units (ICU), wards and home environments to evaluate respiratory muscle function, to titrate ventilatory support levels and to guide recovery from acute illness [1–6]. Obtaining direct recordings of neuron action potentials of the respiratory centers in the human brainstem is impossible. Therefore, provided that phrenic nerve transmission is intact, and the diaphragm is used as the primary inspiratory muscle, the electrical activation of the diaphragm is considered the closest available surrogate to infer the strength and timing of neural respiratory drive [7–9].

The reference standard to measure the electrical activity of the diaphragm (EMGdi) is by using a nasogastric catheter mounted with electrodes on the tip [10]. However, the invasive nature of this technique carries unwanted risks, causes discomfort in spontaneously breathing individuals, and is unsuitable for patients with impaired swallowing function and for those receiving domiciliary ventilation.

Surface electromyography (sEMG) acquired by electrodes such as those used for measurement of the electrocardiogram (ECG) enables transcutaneous measurement of electrical activity of the respiratory muscles. This approach facilitates non-invasive monitoring of respiratory muscles beyond the diaphragm, including the parasternal, sternocleidomastoid, abdominal and scalene muscles [11]. However, the use of respiratory sEMG is still limited in clinical practice. Specific expertise and consensus are required for correct signal acquisition and processing. Additionally, deeper knowledge on its validity and clinical relevance is required [10]. Although general best practices for sEMG acquisition are provided by the ‘Consensus for experimental design in electromyography’ (CEDE) project [12] and the ‘Surface ElectroMyoGraphy for the Non-Invasive Assessment of Muscles’ (SENIAM) initiative [13], specific considerations for respiratory sEMG are lacking. In addition, signal processing can be time-consuming and difficult due to variable measurement setups and strong crosstalk from the heart and adjacent muscles. Nevertheless, due to its non-invasive nature and clinical rationale for respiratory muscle monitoring in the ventilated patient [1], respiratory sEMG popularity is increasing in clinical research worldwide [14]. However, the various approaches to signal acquisition, processing, and interpretation [15, 16] could hinder research comparability and successful, widespread clinical implementation.

### Toward consensus

A 4-day expert roundtable was held in spring 2023 to discuss the state-of-the-art, challenges and future directions of respiratory sEMG. The expert group was composed of medical doctors, technical physicians, software engineers and biomedical engineers experienced in respiratory sEMG and working in the acute (ICU) and/or chronic care (home mechanical ventilation) setting (see Additional file 1 for more details). This paper provides recommendations for state-of-the-art acquisition, processing and interpretation of respiratory sEMG. Additionally, it addresses challenges and explores potential clinical applications of respiratory sEMG in patients with respiratory failure. The overarching objective is to advocate for the standardization and generalizability of respiratory sEMG in clinical research.

## Acquisition

Respiratory sEMG acquisition entails all activities needed to obtain the digitized raw sEMG signal. Table 1 summarizes recommended electrode positions for the most studied respiratory muscles. Legitimate reasons could exist to deviate from these recommendations, such as practical constraints imposed by a clinical or research setup. Skin preparation, e.g., shaving, cleansing and scrubbing, is advised to optimize the sEMG recording quality, as it can improve the signal-to-noise ratio and reduce contaminations. Practical recommendations for skin preparation, electrode type and size are outlined in general sEMG guidelines [12, 13].

**Table 1:** Recommended electrode positions (MCL: Midclavicular Line, AAL: Anterior Axillary Line, ICS: Intercostal Space, MAL: Mid-Axillary Line)

Muscle	Options	Electrode 1 (Anode)	Electrode 2 (Cathode)
Diaphragm	Bilaterally long Unilaterally long Unilaterally short	MCL subcostal Left Xiphoid AAL 6th/7th/8th ICS Right	MCL subcostal Right MAL subcostal Right AAL 7th/8th/9th ICS Right
Parasternal	Bilaterally Unilaterally	2nd/3rd ICS Left 2nd rib, sternal edge	2nd/3rd ICS Right 3rd rib, 2 cm lateral to sternal edge
Sternocleidomastoid	Mastoid/Clavicular notch	Lower 1/3 portion, 2 cm apart	
Scalene	Posterior triangle of the neck at the level of the cricoid process		
Ext. oblique	Combined	AAL, 1/2 costal margin à Iliac crest	MCL medially from anode
Int. oblique			

### Electrode positioning

**Fig. 1:**
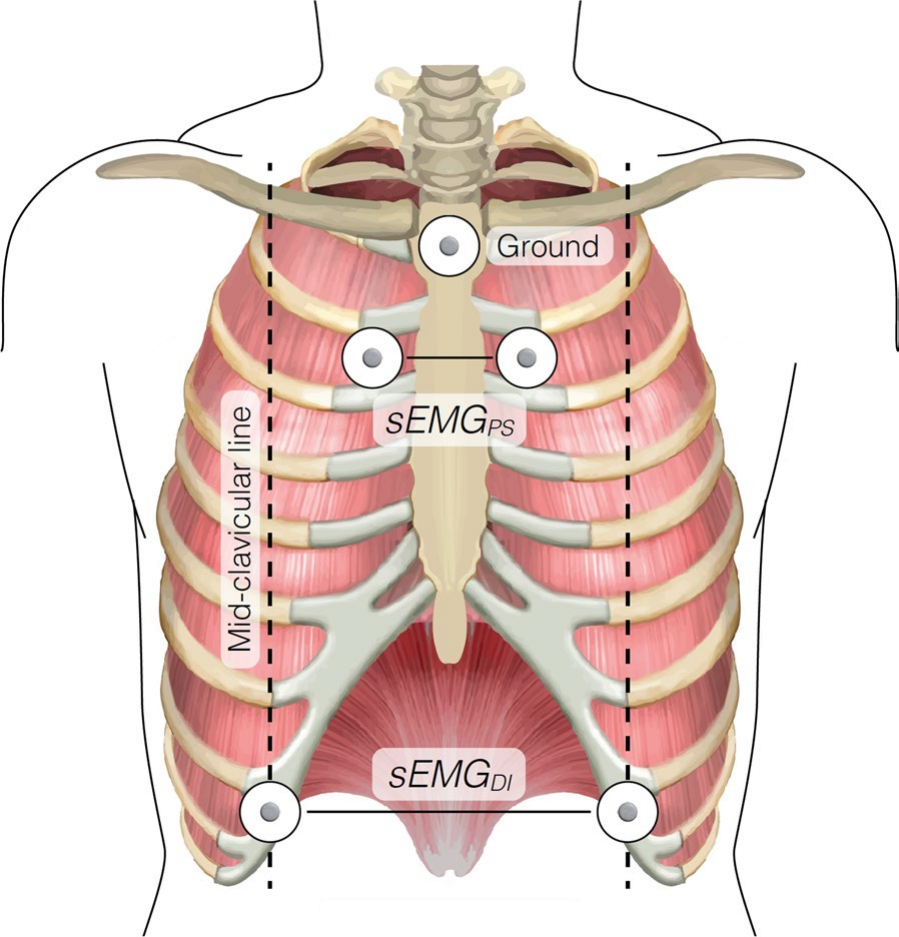
Electrode positioning for bilateral parasternal (sEMG_PS_) and diaphragm (sEMG_DI_) configuration, redrawn from [107]

Pinpointing a one-size-fits-all approach for acquiring diaphragm sEMG is complicated, specifically due to the muscle’s dome shape that is strongly affected by patient positioning, lung and thoracic mechanics, as well as intrathoracic and intra-abdominal pressures [17]. Moreover, other muscles including the abdominal external oblique muscle and the intercostal muscles overlay the diaphragm [18]. Ultrasound may be used to detect specific pathologies that may affect electrode placement, like unilateral acquisition of diaphragm activity in the presence of a diaphragm hemiparesis. Furthermore, ultrasound can be used to guide and validate electrode position in relation to the muscle belly [11, 19]. An end-expiratory occlusion test, or in cooperative patients a sniff or maximal inspiratory maneuver, can be used for this purpose as well. When unilateral diaphragm pathology is not expected, it is advised to acquire diaphragm sEMG from the bilateral configuration (Fig. 1). When unilateral pathology is expected or specific information of one hemidiaphragm is required, a unilateral configuration should be used, possibly on both sides. Unilateral configuration can be obtained either with long or short interelectrode distance, see Table 1.

sEMG of extra-diaphragmatic respiratory muscles can also be acquired using bilateral configurations, although adequate electrode positioning over these often small and short muscle bellies can be challenging in clinical practice. Parasternal sEMG can be obtained with electrode positioning over the second intercostal space, as these intercostal spaces show the least postural artifact during breathing, and the amount of subcutaneous fat is relatively limited [20]. However, crosstalk effects complicate analysis and interpretation; they depend on the intercostal space the muscle is located, the exact origins and insertions onto the respective ribs, the moment in the respiratory cycle, and the amount of lung inflation. Parasternal intercostal muscles located in a more cranial intercostal space will activate earlier and to a greater extent, i.e., the neural drive is coupled to mechanical advantage [21]. Scalene and sternocleidomastoid electrode positioning over the lower portion of each muscle is recommended (adapted from [19] and [22]).

The expiratory abdominal muscles (i.e., rectus abdominis, internal and external oblique and transversal abdominal muscles) can strongly interact with inspiratory muscle action [21]. Considering that crosstalk from the abdominal muscles is common in diaphragmatic sEMG [18, 23], acquisition of abdominal sEMG as a separate channel is advised. This could enable identification of expiratory abdominal muscle crosstalk in the diaphragm leads, thereby facilitating analysis and interpretation of inspiratory diaphragm activity. Crosstalk could be detected by visual inspection. Importantly, when exact timing differences between muscle activation in the diaphragm and expiratory muscle sEMG leads are of interest, using a similar preprocessing pipeline for both signals is recommended (see Sect. “Preprocessing”).

The ground electrode is advised to be placed on a bony structure such as the sternum or clavicle, but its exact location is not expected to significantly affect the acquired signal.

### Interelectrode distance

The distance between electrodes determines the pick-up area and thus affects both the amplitude and frequency characteristics of the resultant signal. Since the diaphragm extends over a large area and deeply into the torso, an electrode configuration with a relatively large interelectrode distance, such as the bilateral configuration, is more likely to capture most of the diaphragm’s activity [24]. It should be noted that large interelectrode distance comes at the cost of increasing muscle crosstalk.

### Technical considerations

The sEMG signal should be acquired at a sampling frequency (f_s_) of at least 500 Hz, ideally 1000 Hz, because the spectral content of respiratory EMG mainly ranges between 25 and 250 Hz [25, 26]. To eliminate baseline wander, a 0.1 Hz high-pass filter is advised as well as an antialiasing low-pass filter. To note, if an acquisition device applies analogue filtering, reporting these settings is advised.

In addition, it is recommended to synchronously acquire auxiliary measures of breathing activity, such as pressure, flow, or volume, to be able to differentiate between inspiratory and expiratory activity, and to trace and decontaminate from any artifacts.

## Preprocessing

**Fig. 2:**
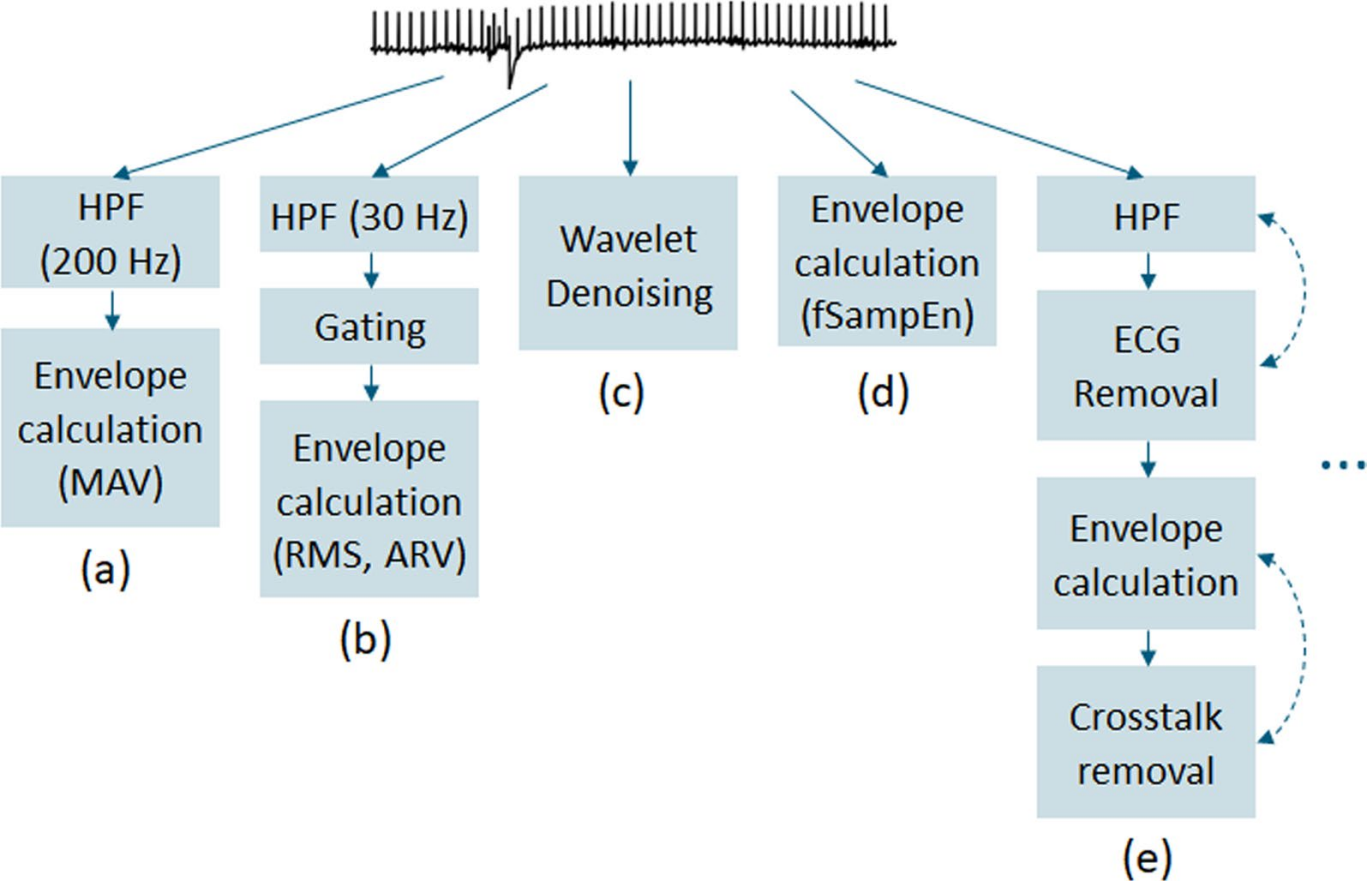
Representation of five potential sEMG preprocessing pipelines composed of different basic building blocks. The most appropriate pipeline to use will depend on the target application scenario and may differ from the ones shown. **a** Simple pipeline for data checks. **b** Gating pipeline for strong ECG interference when EMG amplitudes are to be maintained. **c** Wavelet denoising as the go-to method in most cases (e.g., for raw EMG analyses). **d** Fixed sample entropy for robust envelope calculation without additional ECG removal. **e** Illustration of advanced preprocessing pipeline comprising multiple iterations of ECG removal and crosstalk removal

Raw respiratory sEMG is contaminated by a variety of noise types, complicating the interpretation of the neural activation duration and amplitude. By sEMG preprocessing, we refer to all activities for noise and artifact removal, as well as smoothing, to prepare the signal for parameter calculation. We provide basic building blocks for designing respiratory sEMG preprocessing pipelines (Fig. 2) according to the clinical and research goals. Table 2 provides specific characteristics, pitfalls, and best practices of these building blocks. It is crucial to consider that every filter step alters the frequency spectrum, amplitudes, and timing components of the sEMG, which can be critical if the parameter of interest strongly depends on such characteristics. Comprehensively reporting the applied preprocessing steps thus promotes the reproducibility and generalizability of research.

**Table 2:** Preprocessing

	Specifics	Common pitfalls	Best practice
*ECG removal*			
High-pass filtering	Specific for first ‘raw’ data checks and preliminary step for some preprocessing methods (gating) The higher the ratio between respiratory and cardiac signal power, the lower the required cutoff frequency to reduce impact of cardiac activity on the filtered signal (such as in small-distance electrode setups and parasternal EMG when muscle activation is strong	Distortion of the spectrum Reduction in EMG amplitude Absence of respiratory EMG amplitude in the filtered signal when there is cardiac interference and/or very weak respiratory muscle activation	If used as the sole method, a high cutoff frequency should be employed and adjusted to minimize impact of cardiac activity Mean Absolute Value (MAV) is recommended to obtain the respiratory waveform in cases where QRS peaks are still present in the resulting signal Lower cutoff frequencies (< 50Hz) should be combined with other preprocessing techniques to fully remove the QRS complex
Gating	Envelope calculation when EMG amplitude is to be maintained Requires robust detection of R-peaks	Cannot be used with tachycardia Substantial loss of temporal information Not suitable for detecting respiratory onset/offset with high precision	Pan-Tompkins algorithm should be used to detect R-peaks Combination with 20 Hz high-pass filter to remove P and T waves Window length should be adjusted to the duration of the QRS complex Appropriate gate-filling techniques must be used (interpolation or median)
Wavelet	Go-to method for ECG removal in far-distance electrode setups (with strong ECG interference) when resp muscle activation is small Best method when R-peaks cannot be robustly detected (e.g., many ectopic beats, patients with arrhythmias)	Inadequate setting of Fs, level of decomposition, thresholds Thresholding might cutoff large EMG activity bursts	Pre-filtering is not required Number of decomposition levels depends on sampling frequency and should be adjusted to the P-/T- waves and motion artifacts (10–20 Hz): 5 levels for f_s_ of 1000 Hz, increase/decrease level when f_s_ doubles/halves Resulting wavelet-bands and thresholds should be checked visually Daubechies 2 and 4 wavelets have demonstrated good performance in denoising respiratory EMG [29, 35, 36] Fixed threshold; start with a threshold set at 4.5 times the standard deviation of the decomposition level (σk)
*Envelope*			
*General recommendation: Use centered window with length 250 ms, deviate when application demands*			
Root Mean Square (RMS)	Most generally used Power of the signal can be used based on RMS (and compared with that obtained by spectral methods)	N/A	Step size of the moving window should be considered (1 sample step is feasible)
Average Rectified Value (ARV)	Less affected by high amplitude peaks (like remaining QRS artifacts) than RMS		
Mean Absolute Value (MAV)			Combination with HPF
Fixed sample entropy (fSampEn)	More robust than RMS and ARV, i.e., less affected by high amplitude peaks caused by remaining artifacts	Step size of the moving window: 1 sample step can be computationally expensive (for fSampEn)	Application directly to raw data, no other filtering needed Embedded dimension (m = 1) Tolerance value (r) set to 0.2–0.3 times the standard deviation of the sEMG signal

### Low-frequency artifact removal

Classic high-pass filtering (HPF) with a 0.5–20 Hz cutoff frequency is advised to deal with low-frequency artifacts. These artifacts arise from cable or electrode motion, remaining baseline wander, and low-frequency components of the ECG (such as P and T waves) [27]. Power line interference (50 or 60 Hz) can be suppressed by following standard recommendations as described previously [12, 28].

### ECG removal

**Fig. 3:**
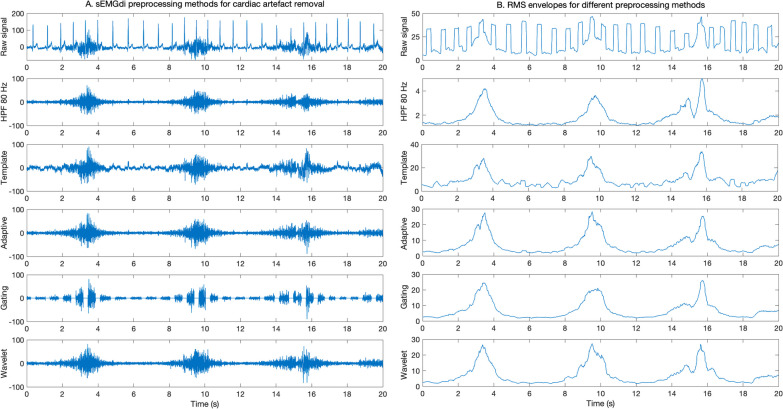
Summary of preprocessing steps for cardiac artefact removal (**A**) and RMS envelopes for the different preprocessing methods (**B**)

Cardiac crosstalk is the primary contaminant of respiratory sEMG, represented by the ECG, often surpassing the sEMG power by orders of magnitude. The substantial overlap in both temporal and spectral domains poses challenges to successful denoising. A variety of algorithmic approaches have been proposed, exploiting different features of the ECG and EMG to solve the separation [15, 16, 29–34]. The complexity of the filtering procedure can be adjusted based on the intended use, also considering the electrode setup and the specific muscle. For instance, analyses of the raw sEMG spectrum would require more advanced filtering techniques, whereas simpler approaches (which typically strongly alter the signal spectrum) often suffice for estimating sEMG amplitudes. Next, best practices for single-channel ECG removal will be discussed. Examples of these methods are provided in Fig. 3A.

A rudimentary approach to removing the ECG artifact from the sEMG signal is HPF with a relatively high cutoff frequency up to 200 Hz [29]. It removes the ECG contamination while preserving the high-frequency components of the sEMG. This approach is best suited for first data checks in setups with small electrode distances, where it gives direct insight into breathing activity. Although this might work well for specific applications, HPF should be used with caution, because it inevitably alters the sEMG spectrum and amplitude.

Another widely used filtering technique is gating, which is conceptually straightforward. The gating technique relies on detecting the QRS complex and removing a small window of samples around it, requiring only a few parameters to be adjusted (Table 2). Gating is robust to very strong ECG interference and does not distort the spectrum or amplitude of the EMG in between the gates [15, 16]. Therefore, it is applicable for analyzing sEMG amplitudes when the interelectrode distance is large, e.g., in the bilateral electrode setup. However, by design, it entails a loss of temporal information, which in some cases requires discarding a substantial part of the data and filling the gates with for example a fixed value or interpolation method [16].

Wavelet denoising is recommended in most cases, including raw sEMG signal analysis, due to its balanced trade-off between implementation complexity and performance [35, 36]. This method is based on decomposition of the signal into several wavelet components and applying a threshold in the wavelet domain to remove ECG interference, and subsequently reconstructing the sEMG signal using the attenuated components. Wavelet denoising is effective when dealing with significant ECG artifacts due to its ability to exploit amplitude differences between the signal (sEMG) and noise (ECG). Properly adjusting the design parameters (see Table 2) before applying wavelet denoising is crucial [29, 35, 36].

Beyond the herein discussed methods, many more cardiac artifact removal algorithms have been described in the literature [16, 22, 29, 33, 37], but their adoption in clinical practice has been limited so far: these methods usually require highly specific and customized adjustment of parameters for each acquired signal, or need a dedicated reference ECG recording [38].

### Envelope signal

The envelope of the sEMG signal reflects the magnitude of the signal over time, providing valuable information about the respiratory muscle activity. After denoising, this demodulated EMG signal can be derived, for example, by calculating the average rectified value (ARV) or the root-mean-square (RMS) over a moving window. Figure 3B shows an example of RMS envelopes for different preprocessing methods. The key parameter to be adjusted when obtaining such an envelope is the window length, being the time frame over which RMS or ARV are calculated. Increasing the window length, for example, will improve the smoothness of the signal at the cost of slower reactivity. The causality of the window (i.e., the dependence of the filter output on past or future inputs) should be guided by the application, as it affects the timing of the signal. For example, the causality of the window could result in incorrect assessment of patient-ventilator asynchronies such as trigger or cycling delays. When cardiac artifacts remain present in the envelope sEMG, visualized as (QRS peak) outliers, more robust amplitude estimators are advised, such as median absolute value (MAV) [29] or fixed sample entropy (fSampEn), which can be applied directly to the raw data even without using any cardiac artifact removal algorithm [39]. Additional file 2 illustrates the effect of these different envelope computation methods.

The respiratory sEMG envelope often has a noticeable offset due to background noise, which is visible between breaths when muscle activation is low. We advise correcting offsets prior to further analyses by subtracting a baseline noise level; however, this level might fluctuate over the duration of the signal, thereby complicating the correction. For RMS envelopes, it is preferable to remove the noise variance instead of the standard deviation [40].

## Postprocessing

Postprocessing is the final stage of signal processing where the parameters of interest are extracted from the decontaminated signal. Key properties that can be computed from the preprocessed sEMG signal (either raw or envelope), their applications and limitations are summarized in Table 3.

**Table 3:** Summary of sEMG parameters and their applications and limitations

Key parameter	Definition/calculation	Potential application/benefits	Notes & limitations
*Magnitude of muscle activity*			
Amplitude	Difference between maximum and minimum value during. one breath (either including or excluding the baseline) Using the 95th and 5th percentile to calculate this difference may be more robust	Assessing changes in absolute magnitude of muscle activity within a single recording	Low amplitude does not imply low muscle activity and vice versa Only comparable within short-time recordings Does not enable between-patient or between-recording comparisons
Amplitude normalized to maximum breathing effort	Amplitude divided by maximum amplitude obtained during maximum inspiratory maneuver	Assessing changes in *relative* muscle activity Improves sEMG amplitude interpretability	Maximum inspiratory maneuvers could be challenging to perform in critically ill patients and multiple repetitions are required Maximum amplitude should be re-obtained for a new recording
Amplitude normalized to maximum amplitude within recording	Amplitude divided by maximum amplitude obtained over a given measurement (without ensuring maximum effort)	Assessing changes in *relative* muscle activity within a patient during a recording Improves sEMG amplitude interpretability	Maximum amplitude should be re-obtained for a new recording It does not enable between-patient comparisons or within-patient comparisons across multiple recordings
EMG-time product	Area under the sEMG envelope, per breath or per time unit	Less sensitive to remaining artifacts than computing breathwise sEMG amplitudes	Dependent on whether the baseline is included in computation Affected by sEMG onset and offset definitions
*Estimation of mechanical output*			
Estimated breathing effort	Pmus = *k* x sEMG, with conversion factor *k* obtained from patient-specific measures (end-expiratory occlusion or model-based)	Translates muscle activity to mechanical output	*k* needs to be re-evaluated for a new recording Assumes a linear relationship between muscle activity and output
*Timing of muscle activity*			
Time-to-peak	Time from onset to peak sEMG	Suggested to reflect respiratory drive	Onset/offset is not binary; no clear definition exists (see text for approaches) Increase in sEMG activity may not be linear Unclear comparability between patients
Duration of muscle activity	Time from sEMG onset to offset	Informs about the duration of muscle activation	Onset/offset is not binary; no clear definition exists (see text for approaches)
Phase angle	Phase lag between sEMG onset (or offset) and start (or end) of ventilator pressurization Phase lag between onset (or offset) of multiple sEMG signals	Assessing patient-ventilator interaction Assessing activation patterns (different muscles)	Onset/offset of sEMG is not binary; no clear definition exists (see text for approaches)
Absolute time delay	Time delay (in ms) between sEMG onset (or offset) and start (or end) of ventilator pressurization Time delay (in ms) between onset (or offset) of multiple sEMG signals	Assessing patient-ventilator interaction Assessing activation patterns (different muscles)	Onset/offset of sEMG is not binary; no clear definition exists (see text for approaches) Does not correct for duration of activity/pressurization such as with phase angle
*Fatigue*			
Fatigue onset	Various metrics have been described: Shift in mean (or median) frequency High/low frequency ratio (*H*/*L* ratio, with *H* = 150–350 Hz and* L* = 20–46.7 Hz) Spectral moments ratio of order five (SMR5) and fuzzy approximate entropy (fApEn)	May inform about diaphragm fatigue before a decrease in pressure-generating capacity occurs	No data and cutoff values exist Challenging to compute reliably in respiratory sEMG due to low SNR

### Magnitude of muscle activity

Ideally, respiratory sEMG amplitude should reflect the magnitude of respiratory muscle activity per breath. Unfortunately, however, this relationship can be disturbed by a range of patient, disease and methodology related factors, e.g., underlying tissue/skin characteristics, fluid balance, end-expiratory lung volume, sweat, electrode type and configuration. Low amplitude despite considerable patient effort could therefore be caused by various confounding factors in signal acquisition, and high amplitude does not necessarily indicate strong muscle activity when artifacts are remaining. Considering the multitude of patient-dependent factors which moreover vary over time, absolute amplitudes will be most comparable within the same patient across a short and stable recording (approximately 30 min). Amplitude comparability over longer recordings or between patients is highly uncertain. To improve robustness to noise, it may be beneficial to determine the breath-wise amplitude using, e.g., the 95th and 5th percentiles, as opposed to the maximum and minimum values. For reference, typically encountered diaphragmatic sEMG amplitudes range between 1 and 10 μV [41–43].

Normalization of sEMG offers means to improve sEMG amplitude interpretability [44] and comparability. Normalization is preferred to a maximal voluntary inspiratory maneuver [45], e.g., by performing an inspiratory capacity maneuver [46] or sniff maneuver [47]. Although maximum inspiratory effort provides a measure of relative muscle activation, maximum-effort inspiratory maneuvers can be challenging to perform. Alternatively, the sEMG signal can be normalized to an EMG signal at a specific ventilatory support level [11]. Normalization to the maximum amplitude over a given measurement could be used to assess relative changes in muscle activation within a recording, e.g., following ventilator adjustments, or after changes of resistances during inspiratory muscle training.

Alternatively, as an estimate of the neural respiratory drive, the area under the inspiratory waveform (i.e., sEMG-time product [42, 48]) reflects the intensity of muscle activation. This measure is less sensitive to remaining artifacts than instantaneous sEMG amplitudes. Nonetheless, it is highly dependent on sEMG onset and offset definitions, and whether the baseline is included in its computation. Moreover, its comparability between patients and over long-term recordings suffers from the same challenges as other amplitude measures.

### Estimation of force generation

Without proper normalization, the above parameters do not reflect force or pressure generation of the muscle. To estimate breathing effort (e.g., Pmus) from sEMG measurements, a conversion factor can be derived from patient-specific measurements. Current methods assume a linear relationship between Pmus and sEMG:

$${\text{Pmus }} = k\; \times {\text{ sEMG}},$$

with *k* the conversion factor and sEMG the peak amplitude of the signal. This conversion factor, also referred to as the neuromechanical efficiency (NME) index, is determined from simultaneously obtained pneumatic measurements [41, 42, 48–50]. This can be done during specific maneuvers, such as end-expiratory occlusions [41, 50], in which case a correction factor of 0.7 or 0.8 is needed as the diaphragm is more efficient during isometric contractions as compared to tidal breathing [41, 42]. Newer model-based approaches use the equation of motion to determine the conversion factor during tidal breathing and computationally derive Pmus [48]. The latter study [48], demonstrated high reliability and accuracy for estimation of Pmus, even for recordings with low sEMG quality, but this should be confirmed in an external cohort of critically ill patients.

Despite these promising studies, there are several methodological considerations with respect to using the NME as a conversion factor between sEMG and Pmus. First, Pmus is the result of the summed effect of all respiratory muscles, and thereby, NME does not resemble a physiologically meaningful efficiency measure of a single respiratory muscle. Second, NME depends on many factors related to its measurement, including thickness of the subcutaneous fat layer, electrode impedance, electrode placement, and signal pre- and postprocessing. Therefore, an sEMG-derived NME is only stable over a short period of time. Third, the relationship between force and EMG is not linear at the extremes of lung volumes considering the diaphragm force–length relationship and its geometry and is influenced by the inverse relationship between force and velocity of muscle contraction [51, 52].

### Timing of muscle activity

Analysis of onset and offset of muscle activity can inform about muscle activation patterns [53] or could quantify patient-ventilator interaction when synchronized pneumatic signals are available [54]. No clear definitions for sEMG onset and offset are available as muscle activation is not a binary process. (Semi-)automated quantification could involve, e.g., a change in the slope of the sEMG envelope signal, reaching a pre-defined threshold, or the crossing of specific thresholds on pneumatic signals [11, 22, 55–59]. Manual detection has also been reported [56, 59–61] but is cumbersome and time-consuming. End of inspiration as defined by a drop to 70% of the peak envelope sEMG is often used [56–58, 62], as well as its return to baseline. The latter may however be hard to determine in clinical practice. The relative timing of any sEMG signal relative to pneumatic data should always be interpreted with caution, since delays and morphological changes could be introduced in any step from data acquisition to envelope extraction of either data type. Future work should focus on developing and validating robust and standardized algorithms for automated detection of timing parameters.

The timing coordination as computed by the phase angle (or absolute time-delay) between sEMG and ventilator pressurization on- and offset has been used to quantify the timing of sEMG onset relative to flow [22, 63] and also various patient-ventilator asynchronies, such as ineffective efforts or reversed triggering [64]. It could also inform about the activation patterns of different respiratory muscles [11]. Assessing the changes in relative timing between the diaphragm and accessory muscles could be an interesting future approach, considering that patients likely shift their respiratory drive to accessory muscles before neuromuscular fatigue of the diaphragm is strongly manifested [5, 21].

### Fatigue assessment

Theoretically, changes in the conversion factor of sEMG to Pmus over time may be indicative of changes in the diaphragm’s force-generating capacity and occurrence of fatigue. However, the short-term stability of this factor *k* may hamper such reliable interpretation. EMG-based approaches have been described to quantify diaphragm fatigue in healthy subjects [65, 66], but limited data exist on the relevance and reliability of these measurements for the respiratory muscles in critically patients. Electrical signs of diaphragm fatigue were reported in 1989, using invasively measured EMG in patients meeting the usual criteria for weaning failure [67]. More recently, spectral changes in invasively measured diaphragm EMG were reported during inspiratory loading [68]. Complex time-domain methods have been proposed for peripheral muscles based on wavelets and entropy [69] or in-depth analysis of spectral densities [70]. Their potential applicability to diaphragmatic sEMG signals has been demonstrated in a simulation study [71], but needs clinical investigation. Importantly, whether fatigue assessment is relevant for patients in the ICU or chronic ventilation setting is at present unclear.

## Applications in research and clinic

Respiratory sEMG has been applied in many clinical situations (Table 4). Neural respiratory drive has been shown to be correlated with dyspnea sensation [72–74], respiratory loading [2, 75, 76], clinical deterioration/exacerbations [77], recovery from exacerbations [4] and even mortality [78]. With increased loading, activation patterns of the diaphragm and accessory respiratory muscles change [11, 76, 79, 80]. Parasternal and scalene activity has been shown to serve as surrogate for general respiratory activity in absence of diaphragm recordings, and high respiratory load changes the correlation between diaphragm and parasternal activity in both adult and pediatric patients [11, 81].

**Table 4:** Clinical applications

Goal	Setting	Use	References
*Investigate mechanisms*			
Investigate mechanisms of respiratory muscle activation	Research	For example: Muscle activation during coughing Respiratory muscle activity in health and disease Respiratory muscle activation during inspiratory loading	[2, 73, 108–115]
Investigate mechanisms of breathlessness	Research	Breathlessness in COPD, during exercise	[72, 76, 116, 117]
*Monitoring disease*			
Diagnostic/Monitor disease severity	ICU/RCU/ward/home	Monitor respiratory muscle activity in pre-school children with airway symptoms	[118, 119]
Predict change in clinical condition	ICU/RCU/ward/home	Monitor respiratory muscle activity to detect recovery and deterioration, need for intervention, post-discharge outcomes	[4, 77, 85]
Predict prognosis	Home	Predict long-term outcomes following AECOPD	[78]
Response to intervention			
Titrate inspiratory muscle training	ICU/RCU/ward/home	Quantify respiratory muscle activation in response to different modalities and resistances	[53, 87, 120]
Response to other interventions	ICU/RCU/ward/home	For example: Response intermittent hypoxia to improve motor plasticity in ALS Response to an arithmetic task in asthmatic children Response of upper airway muscles to non-invasive ventilation	[121–123]
*To optimize mechanical ventilation*			
Titrate mechanical ventilation	ICU/RCU/ward/home	Quantification of inspiratory effort and contribution of the different respiratory muscles in order to define the optimal level of support Detect patient-ventilator asynchrony	[3, 11, 49, 76, 81, 124]
Facilitate weaning from mechanical ventilation	ICU/RCU	Monitor respiratory muscle activity to detect SBT failure	[6]

### Monitoring and prognostic applications

sEMG is an attractive tool to monitor respiratory muscle activation in various diseases. Disease progression and respiratory exacerbations deleteriously affect patients’ symptoms and health-related quality of life and are associated with increased healthcare costs [82–84]. sEMG has been applied during severe COPD exacerbations, with inpatient changes predictive of clinician-defined deterioration, safe discharge without 28-day readmission, and post-discharge recovery and survival [4, 77, 78]. Although patients are limited by being connected to wires potentially impairing their mobilization, sEMG is non-invasive and patient friendly. It can be performed without any patient effort and is an attractive tool to monitor patients in whom monitoring based on effort dependent tests is less suitable or impossible. sEMG has been applied in the impatient pediatric setting, where voluntary lung function testing is challenging, and correlates with clinical asthma scores [85].

### Monitoring during inspiratory muscle training

Inspiratory muscle training (IMT) aims to improve respiratory muscle strength and endurance. Performing sEMG measurements during IMT allows for monitoring of respiratory muscle activation in response to different modalities and resistances [86], potentially enabling clinicians to tailor training characteristics to optimize its results [87] with sEMG parameters as outcome measure [88, 89]. For example, sEMG measurements during exercise have been shown to reflect changes in respiratory muscles activation after IMT that were also associated with reduction in dyspnea sensation [90, 91].

### Optimization of mechanical ventilation

#### In the intensive care unit

Monitoring respiratory muscle activity with sEMG in mechanically ventilated critically ill patients has the potential to provide valuable information for clinicians. In this paragraph, we elaborate on clinical applications that are currently studied, although its clinical utility should be corroborated in larger clinical trials. First, sEMG may be used to titrate the level of support during assisted mechanical ventilation. Multiple studies have shown that accessory muscle activation, as a potential sign of high loading, is correlated with the level of pressure support [11, 92]. Second, sEMG may be used to detect patient-ventilator asynchrony [74, 93]. Third, sEMG of the accessory respiratory muscles may help to assess the response to a spontaneous breathing trial (SBT). Near maximum activation of sternocleidomastoid shortly after the start of the SBT was found in patients with SBT failure and associated with impaired diaphragm activity [94]. Furthermore, expiratory muscle activation was detected in the late phase of the SBT [6]; this can be considered as a compensatory mechanism in the presence of an imbalance between load and capacity of the respiratory muscle pump [18].

#### Home mechanical ventilation

Home mechanical ventilation (HMV) has been shown to improve symptoms, health-related quality of life and admission-free survival in COPD [95–97], obesity hypoventilation syndromes [98] and neuromuscular disease such as amyotrophic lateral sclerosis (ALS) [99, 100]. Although synchronized interaction between the patient and ventilator is known to influence comfort, breathlessness, and sleep quality [101, 102], patient-ventilator asynchrony is common in HMV without adversely impacting upon effective gas exchange [3]. Settings are titrated to optimize gas exchange and patient comfort [103]. However, a proportion of patients does not acclimatize to the ventilator or experience problems when their underlying diseases progresses, leading to suboptimal adherence and therapy efficacy [104]. The utility of sEMG to monitor muscle activation can inform the clinician about patient-ventilator interaction and could provide additional information as compared to inspecting ventilator waveforms and respiratory inductance plethysmography solely [3, 105]. 

## Future perspectives

Respiratory sEMG provides insight in the electrical activation of the respiratory muscle pump, similar to how the ECG reflects cardiac function, and can be used to support clinical assessment and decision making in a range of settings and patient cohorts. Although the many potential applications, implementation of sEMG remains challenging. To facilitate its widespread use into routine clinical practice, standardized acquisition and presentation of respiratory sEMG outcomes are of utmost importance; the need for accuracy and transparency in reporting of the applied hardware and software methods should not be underestimated, and researchers should be stimulated to publish their precise method and code. Further development of open-source and transparent software for analysis and interpretation, as for example ReSurfEMG [106], is encouraged. Clinicians should be aware that respiratory sEMG is dynamic and that guidance on how to best indicate the stability and reliability of the recordings needs to be further developed. This will also allow clinicians to study and understand the best parameters and cutoff values that indicate important clinical changes. The clinical applications of sEMG are numerous, yet further standardization of the technology will enable and stimulate its routine use.

## Conclusion

Monitoring of patients with respiratory difficulties provides information that may facilitate early intervention, prevent deterioration and (ICU) hospitalization. Respiratory sEMG is a noninvasive tool for respiratory monitoring, but widespread implementation is hindered by practical challenges and pitfalls for acquisition, pre- and postprocessing. This paper outlines important clinical and technological considerations and provides best-practice recommendations for different uses, from acute critical care to the home setting. This is key for further development and implementation of respiratory sEMG.

### Supplementary Information

**Additional file 1**. AF1_Background_expert_group. A comprehensive overview of the background, clinical field and academic degree of the expert group

**Additional file 2**. AF2_Comparison_envelope. A visual overview multiple methods for envelope calculation

## Data Availability

Not applicable.

## References

[1] Doorduin J, Van Hees HWH, Van Der Hoeven JG, Heunks LMA (2013). Monitoring of the respiratory muscles in the critically ill. Am J Respir Crit Care Med.

[2] Duiverman ML, Van Eykern LA, Vennik PW, Koëter GH, Maarsingh EJW, Wijkstra PJ (2004). Reproducibility and responsiveness of a noninvasive EMG technique of the respiratory muscles in COPD patients and in healthy subjects. J Appl Physiol.

[3] Ramsay M, Mandal S, Suh ES, Steier J, Douiri A, Murphy PB, Polkey M, Simonds A, Hart N (2015). Parasternal electromyography to determine the relationship between patient-ventilator asynchrony and nocturnal gas exchange during home mechanical ventilation set-up. Thorax.

[4] D’Cruz RF, Suh E-S, Kaltsakas G, Dewar A, Shah NM, Priori R, Douiri A, Rose L, Hart N, Murphy PB (2021). Home parasternal electromyography tracks patient-reported and physiological measures of recovery from severe COPD exacerbation. ERJ Open Res.

[5] Dres M, Demoule A (2018). Diaphragm dysfunction during weaning from mechanical ventilation: an underestimated phenomenon with clinical implications. Crit Care.

[6] Pozzi M, Rezoagli E, Bronco A, Rabboni F, Grasselli G, Foti G, Bellani G (2022). Accessory and expiratory muscles activation during spontaneous breathing trial: a physiological study by surface electromyography. Front Med (Lausanne).

[7] Sinderby C, Navalesi P, Beck J, Skrobik Y, Comtois N, Friberg S, Gottfried SB, Lindström L (1999). Neural control of mechanical ventilation in respiratory failure. Nat Med.

[8] Tobin M, Gardner W, Tobin M (1998). Monitoring the control of breathing. Principles and practice of intensive care monitoring.

[9] Telias I, Spadaro S (2020). Techniques to monitor respiratory drive and inspiratory effort. Curr Opin Crit Care.

[10] American Thoracic Society/European Respiratory Society (2002). ATS/ERS Statement on respiratory muscle testing. Am J Respir Crit Care Med.

[11] Roesthuis LH, van der Hoeven JG, van Hees HWH, Schellekens WJM, Doorduin J, Heunks LMA (2020). Recruitment pattern of the diaphragm and extradiaphragmatic inspiratory muscles in response to different levels of pressure support. Ann Intensive Care.

[12] CEDE Project | International Society of Electrophysiology and Kinesiology (ISEK). https://isek.org/cede-project/. Accessed 30 Jun 2023

[13] www.seniam.org.

[14] da Silva Junior EFF, Campos SL, Leite WS, de Sousa Melo PV, Lins RAC, AraújoGuerino MDMR (2023). Surface electromyography signal processing and evaluation on respiratory muscles of critically ill patients: a systematic review. PLoS One.

[15] Hutten GJ, Van Thuijl HF, Van Bellegem ACM, Van Eykern LA, Van Aalderen WMC (2010). A literature review of the methodology of EMG recordings of the diaphragm. J Electromyogr Kinesiol.

[16] van Leuteren RW, Hutten GJ, de Waal CG, Dixon P, van Kaam AH, de Jongh FH (2019). Processing transcutaneous electromyography measurements of respiratory muscles, a review of analysis techniques. J Electromyogr Kinesiol.

[17] Suwatanapongched T, Gierada DS, Slone RM, Pilgram TK, Tuteur PG (2003). Variation in diaphragm position and shape in adults with normal pulmonary function. Chest.

[18] Shi ZH, Jonkman A, de Vries H, Jansen D, Ottenheijm C, Girbes A, Spoelstra-de Man A, Zhou JX, Brochard L, Heunks L (2019). Expiratory muscle dysfunction in critically ill patients: towards improved understanding. Intensive Care Med.

[19] Falla D, Dall’Alba P, Rainoldi A, Merletti R, Jull G (2002). Location of innervation zones of sternocleidomastoid and scalene muscles—a basis for clinical and research electromyography applications. Clin Neurophysiol.

[20] Wallbridge P, Parry SM, Das S, Law C, Hammerschlag G, Irving L, Hew M, Steinfort D (2018). Parasternal intercostal muscle ultrasound in chronic obstructive pulmonary disease correlates with spirometric severity. Sci Rep.

[21] De Troyer A, Boriek AM (2011). Mechanics of the respiratory muscles. Compr Physiol.

[22] Rodrigues A, Janssens L, Langer D, Matsumura U, Rozenberg D, Brochard L, Reid WD (2022). Semi-automated detection of the timing of respiratory muscle activity: validation and first application. Front Physiol.

[23] Sinderby C, Friberg S, Comtois N, Grassino A (1996). Chest wall muscle cross talk in canine costal diaphragm electromyogram. J Appl Physiol.

[24] Merletti R, Muceli S (2019). Tutorial. Surface EMG detection in space and time: best practices. J Electromyogr Kinesiol.

[25] Schweitzer TW, Fitzgerald JW, Bowden JA, Lynne-Davies P (1979). Spectral analysis of human inspiratory diaphragmatic electromyograms. J Appl Physiol.

[26] Van Boxtel A, Boelhouwer AJW, Bos AR (1998). Optimal EMG signal bandwidth and interelectrode distance for the recording of acoustic, electrocutaneous, and photic blink reflexes. Psychophysiology.

[27] Thakor NV, Webster JG, Tompkins WJ (1984). Estimation of qrs complex power spectra for design of a QRS filter. IEEE Trans Biomed Eng BME.

[28] Stegeman D, HH-ERR. Standards for surface electromyography: The European project Surface EMG for non-invasive assessment of muscles (SENIAM). Citeseer (2007)

[29] Petersen E, Sauer J, Grabhoff J, Rostalski P (2020). removing cardiac artifacts from single-channel respiratory electromyograms. IEEE Access.

[30] Lu G, Brittain JS, Holland P, Yianni J, Green AL, Stein JF, Aziz TZ, Wang S (2009). Removing ECG noise from surface EMG signals using adaptive filtering. Neurosci Lett.

[31] Willigenburg NW, Daffertshofer A, Kingma I, van Dieën JH (2012). Removing ECG contamination from EMG recordings: a comparison of ICA-based and other filtering procedures. J Electromyogr Kinesiol.

[32] Taelman J, Van Huffel S, Spaepen A (2007) Wavelet-independent component analysis to remove electrocardiography contamination in surface electromyography. In: Annual international conference of the IEEE engineering in medicine and biology - proceedings 682–685 10.1109/IEMBS.2007.4352382 18002048

[33] Sameni R, Shamsollahi MB, Jutten C, Clifford GD (2007). A nonlinear Bayesian filtering framework for ECG denoising. IEEE Trans Biomed Eng.

[34] Github ECG removal. https://github.com/ime-luebeck/ecg-removal. Accessed 18 Sep 2023

[35] Zhan C, Yeung LF, Yang Z (2010). A wavelet-based adaptive filter for removing ECG interference in EMGdi signals. J Electromyogr Kinesiol.

[36] Jonkman AH, Juffermans R, Doorduin J, Heunks LMA, Harlaar J (2021). Estimated ECG Subtraction method for removing ECG artifacts in esophageal recordings of diaphragm EMG. Biomed Signal Process Control.

[37] McSharry PE, Clifford GD, Tarassenko L, Smith LA (2003). A dynamical model for generating synthetic electrocardiogram signals. IEEE Trans Biomed Eng.

[38] Dacha S, Janssens L, Rodrigues A, Louvaris Z, Janssens L, Gosselink R, Langer D (2019). Comparison between manual and (semi-) automated analyses of esophageal diaphragm electromyography during endurance cycling in patients with COPD. Front Physiol.

[39] Estrada L, Torres A, Sarlabous L, Jané R (2016). Improvement in neural respiratory drive estimation from diaphragm electromyographic signals using fixed sample entropy. IEEE J Biomed Health Inform.

[40] Clancy EA, Morin EL, Merletti R (2002). Sampling, noise-reduction and amplitude estimation issues in surface electromyography. J Electromyogr Kinesiol.

[41] Bellani G, Bronco A, Arrigoni Marocco S (2018). Measurement of diaphragmatic electrical activity by surface electromyography in intubated subjects and its relationship with inspiratory effort. Respir Care.

[42] Graßhoff J, Petersen E, Farquharson F, Kustermann M, Kabitz H-J, Rostalski P, Walterspacher S (2021). Surface EMG-based quantification of inspiratory effort: a quantitative comparison with Pes. Crit Care.

[43] Lokin JLC, Dulger S, Glas GJ, Horn J (2020). Transesophageal versus surface electromyography of the diaphragm in ventilated subjects. Respir Care.

[44] Frey Law L, Krishnan C, Avin K (2011). Modeling nonlinear errors in surface electromyography due to baseline noise: a new methodology. J Biomech.

[45] Sinderby C, Beck J, Spahija J, Weinberg J, Grassino A (1998). Voluntary activation of the human diaphragm in health and disease. J Appl Physiol.

[46] Ramsook AH, Molgat-Seon Y, Schaeffer MR, Wilkie SS, Camp PG, Reid WD, Romer LM, Guenette JA (2017). Effects of inspiratory muscle training on respiratory muscle electromyography and dyspnea during exercise in healthy men. J Appl Physiol.

[47] Murphy PB, Kumar A, Reilly C (2011). Neural respiratory drive as a physiological biomarker to monitor change during acute exacerbations of COPD. Thorax.

[48] Graßhoff J, Petersen E, Walterspacher S, Rostalski P (2023). Model-based estimation of inspiratory effort using surface EMG. IEEE Trans Biomed Eng.

[49] Petersen E, Graßhoff J, Eger M, Rostalski P (2020). Surface EMG-based estimation of breathing effort for neurally adjusted ventilation control. IFAC-PapersOnLine.

[50] Jansen D, Jonkman AH, Roesthuis L, Gadgil S, Van Der Hoeven JG, Scheffer GJJ, Girbes A, Doorduin J, Sinderby CS, Heunks LMA (2018). Estimation of the diaphragm neuromuscular efficiency index in mechanically ventilated critically ill patients. Crit Care.

[51] De Troyer A (1997). Effect of hyperinflation on the diaphragm. Eur Respir J.

[52] Finucane KE, Panizza JA, Singh B (2005). Efficiency of the normal human diaphragm with hyperinflation. J Appl Physiol.

[53] Walterspacher S, Pietsch F, Walker DJ, Röcker K, Kabitz HJ (2018). Activation of respiratory muscles during respiratory muscle training. Respir Physiol Neurobiol.

[54] Vignaux L, Vargas F, Roeseler J, Tassaux D, Thille AW, Kossowsky MP, Brochard L, Jolliet P (2009). Patient-ventilator asynchrony during non-invasive ventilation for acute respiratory failure: a multicenter study. Intensive Care Med.

[55] Estrada L, Sarlabous L, Lozano-Garcia M, Jane R, Torres A (2019). Neural offset time evaluation in surface respiratory signals during controlled respiration. Annu Int Conf IEEE Eng Med Biol Soc.

[56] Sinderby C, Liu S, Colombo D, Camarotta G, Slutsky AS, Navalesi P, Beck J (2013). An automated and standardized neural index to quantify patient-ventilator interaction. Crit Care.

[57] Liu L, Xia F, Yang Y, Longhini F, Navalesi P, Beck J, Sinderby C, Qiu H (2015). Neural versus pneumatic control of pressure support in patients with chronic obstructive pulmonary diseases at different levels of positive end expiratory pressure: a physiological study. Crit Care.

[58] Colombo D, Cammarota G, Bergamaschi V, De Lucia M, Della CF, Navalesi P (2008). Physiologic response to varying levels of pressure support and neurally adjusted ventilatory assist in patients with acute respiratory failure. Intensive Care Med.

[59] Koopman AA, Blokpoel RGT, van Eykern LA, de Jongh FHC, Burgerhof JGM, Kneyber MCJ (2018). Transcutaneous electromyographic respiratory muscle recordings to quantify patient–ventilator interaction in mechanically ventilated children. Ann Intensive Care.

[60] Hudson AL, Gandevia SC, Butler JE (2011). Common rostrocaudal gradient of output from human intercostal motoneurones during voluntary and automatic breathing. Respir Physiol Neurobiol.

[61] Epiu I, Gandevia SC, Boswell-Ruys CL, Basha C, Archer SNJ, Butler JE, Hudson AL (2021). Inspiratory muscle responses to sudden airway occlusion in chronic obstructive pulmonary disease. J Appl Physiol.

[62] Estrada L, Torres A, Sarlabous L, Jane R (2018). Onset and offset estimation of the neural inspiratory time in surface diaphragm electromyography: a pilot study in healthy subjects. IEEE J Biomed Health Inform.

[63] Nguyen DAT, Amirjani N, McCaughey EJ, Gandevia SC, Butler JE, Hudson AL (2020). Differential activation of the human costal and crural diaphragm during voluntary and involuntary breaths. J Appl Physiol.

[64] Akoumianaki E, Lyazidi A, Rey N, Matamis D, Perez-Martinez N, Giraud R, Mancebo J, Brochard L, Richard JCM (2013). Mechanical ventilation-induced reverse-triggered breaths: a frequently unrecognized form of neuromechanical coupling. Chest.

[65] Gross D, Grassino A, Ross WRD, Macklem PT (1979). Electromyogram pattern of diaphragmatic fatigue. J Appl Physiol.

[66] Zwarts MJ, Bleijenberg G, Van Engelen BGM (2007). Clinical neurophysiology of fatigue. Clin Neurophysiol.

[67] Brochard L, Harf A, Lorino H, Lemaire F (1989). Inspiratory pressure support prevents diaphragmatic fatigue during weaning from mechanical ventilation. Am Rev Respir Dis.

[68] Doorduin J, Sinderby CA, Beck J, Stegeman DF, Van Hees HWH, Van Der Hoeven JG, Heunks LMA (2012). The calcium sensitizer levosimendan improves human diaphragm function. Am J Respir Crit Care Med.

[69] Kahl L, Hofmann UG (2016). Comparison of algorithms to quantify muscle fatigue in upper limb muscles based on sEMG signals. Med Eng Phys.

[70] Dimitrov GV, Arabadzhiev TI, Mileva KN, Bowtell JL, Crichton N, Dimitrova NA (2006). Muscle fatigue during dynamic contractions assessed by new spectral indices. Med Sci Sports Exerc.

[71] Kahl L, Hofmann UG (2021). Removal of ecg artifacts affects respiratory muscle fatigue detection (A simulation study). Sensors.

[72] Georges M, Moraviec E, Raux M, Gonzalez-Bermejo J, Pradat PF, Similowski T, Morélot-Panzini C (2016). Cortical drive to breathe in amyotrophic lateral sclerosis: a dyspnoea-worsening defence?. Eur Respir J.

[73] McKenzie DK, Butler JE, Gandevia SC (2009). Respiratory muscle function and activation in chronic obstructive pulmonary disease. J Appl Physiol.

[74] Schmidt M, Kindler F, Gottfried SB, Raux M, Hug F, Similowski T, Demoule A (2013). Dyspnea and surface inspiratory electromyograms in mechanically ventilated patients. Intensive Care Med.

[75] Cavalcanti JD, Fregonezi GAF, Sarmento AJ, Bezerra T, Gualdi LP, Pennati F, Aliverti A, Resqueti VR (2022). Electrical activity and fatigue of respiratory and locomotor muscles in obstructive respiratory diseases during field walking test. PLoS One.

[76] Duiverman ML, de Boer EWJ, van Eykern LA, de Greef MHG, Jansen DF, Wempe JB, Kerstjens HAM, Wijkstra PJ (2009). Respiratory muscle activity and dyspnea during exercise in chronic obstructive pulmonary disease. Respir Physiol Neurobiol.

[77] Suh ES, Mandal S, Harding R (2015). Neural respiratory drive predicts clinical deterioration and safe discharge in exacerbations of COPD. Thorax.

[78] Patout M, Meira L, D’Cruz R, Lhuillier E, Kaltsakas G, Arbane G, Suh ES, Hart N, Murphy PB (2019). Neural respiratory drive predicts long-term outcome following admission for exacerbation of COPD: a post hoc analysis. Thorax.

[79] Dres M, Similowski T, Goligher EC (2021). Dyspnoea and respiratory muscle ultrasound to predict extubation failure. Eur Respir J.

[80] Domnik NJ, Phillips DB, James MD (2022). Compensatory responses to increased mechanical abnormalities in COPD during sleep. Eur J Appl Physiol.

[81] Koopman AA, van Dijk J, Oppersma E, Blokpoel RGT, Kneyber MCJ (2023). Surface electromyography to quantify neuro-respiratory drive and neuro-mechanical coupling in mechanically ventilated children. Respir Res.

[82] Suissa S, Dell’Aniello S, Ernst P (2012). Long-term natural history of chronic obstructive pulmonary disease: severe exacerbations and mortality. Thorax.

[83] Niewoehner DE (2006). The impact of severe exacerbations on quality of life and the clinical course of chronic obstructive pulmonary disease. Am J Med.

[84] Hazenberg A, Kerstjens HAM, Prins SCL, Vermeulen KM, Wijkstra PJ (2016). Is chronic ventilatory support really effective in patients with amyotrophic lateral sclerosis?. J Neurol.

[85] Maarsingh EJW, Oud M, Van Eykern LA, Hoekstra MO, Van Aalderen WMC (2006). Electromyographic monitoring of respiratory muscle activity in dyspneic infants and toddlers. Respir Physiol Neurobiol.

[86] Rodrigues A, Louvaris Z, Dacha S, Janssens WIM, Pitta F, Vogiatzis I, Gosselink RIK, Langer D (2020). Differences in respiratory muscle responses to hyperpnea or loaded breathing in COPD. Med Sci Sports Exerc.

[87] Ramsook AH, Koo R, Molgat-Seon Y, Dominelli PB, Syed N, Ryerson CJ, Sheel AW, Guenette JA (2016). Diaphragm recruitment increases during a bout of targeted inspiratory muscle training. Med Sci Sports Exerc.

[88] Lee CT, Chien JY, Hsu MJ, Wu HD, Wang LY (2021). Inspiratory muscle activation during inspiratory muscle training in patients with COPD. Respir Med.

[89] Ando R, Ohya T, Kusanagi K, Koizumi J, Ohnuma H, Katayama K, Suzuki Y (2020). Effect of inspiratory resistive training on diaphragm shear modulus and accessory inspiratory muscle activation. Appl Physiol Nutr Metab.

[90] Pereira MC, Dacha S, Testelmans D, Gosselink R, Langer D (2019). Assessing the effects of inspiratory muscle training in a patient with unilateral diaphragm dysfunction. Breathe.

[91] Schaeffer MR, Louvaris Z, Rodrigues A (2023). Effects of inspiratory muscle training on exertional breathlessness in patients with unilateral diaphragm dysfunction: a randomised trial. ERJ Open Res.

[92] Cecchini J, Schmidt M, Demoule A, Similowski T (2014). Increased diaphragmatic contribution to inspiratory effort during neurally adjusted ventilatory assistance versus pressure support: an electromyographic study. Anesthesiology.

[93] Schmidt M, Chiti L, Hug F, Demoule A, Similowski T (2011). Surface electromyogram of inspiratory muscles: a possible routine monitoring tool in the intensive care unit. Br J Anaesth.

[94] Parthasarathy S, Jubran A, Laghi F, Tobin MJ (1985). Sternomastoid, rib cage, and expiratory muscle activity during weaning failure. J Appl Physiol.

[95] Raveling T, Vonk J, Struik FM, Goldstein R, Kerstjens HAM, Wijkstra PJ, Duiverman ML (2021). Chronic non-invasive ventilation for chronic obstructive pulmonary disease. Cochrane Database Syst Rev.

[96] Ergan B, Oczkowski S, Rochwerg B (2019). European Respiratory Society guidelines on long-term home non-invasive ventilation for management of COPD. Eur Respir J.

[97] Murphy PB, Rehal S, Arbane G (2017). Effect of home noninvasive ventilation with oxygen therapy vs oxygen therapy alone on hospital readmission or death after an acute COPD exacerbation: a randomized clinical trial. JAMA.

[98] Mokhlesi B, Masa JF, Afshar M (2019). Evaluation and management of obesity hypoventilation syndrome. An Official American Thoracic Society Clinical Practice Guideline. Am J Respir Crit Care Med.

[99] Khan A, Frazer-Green L, Amin R (2023). Respiratory management of patients with neuromuscular weakness: an american college of chest physicians clinical practice guideline and expert panel report. Chest.

[100] Bourke SC, Bullock RE, Williams TL, Shaw PJ, Gibson GJ (2003). Noninvasive ventilation in ALS: indications and effect on quality of life. Neurology.

[101] Crescimanno G, Canino M, Marrone O (2012). Asynchronies and sleep disruption in neuromuscular patients under home noninvasive ventilation. Respir Med.

[102] Adler D, Perrig S, Takahashi H, Espa F, Rodenstein D, Pépin JL, Janssens JP (2012). Polysomnography in stable COPD under non-invasive ventilation to reduce patient-ventilator asynchrony and morning breathlessness. Sleep Breath.

[103] Patout M, Arbane G, Cuvelier A, Muir JF, Hart N, Murphy PB (2019). Polysomnography versus limited respiratory monitoring and nurse-led titration to optimise non-invasive ventilation set-up: a pilot randomised clinical trial. Thorax.

[104] Valko L, Baglyas S, Gyarmathy VA, Gal J, Lorx A (2020). Home mechanical ventilation: quality of life patterns after six months of treatment. BMC Pulm Med.

[105] Haynes JM (2017). Patient-ventilator asynchrony and standard waveforms: looks can be deceiving. Respir Care.

[106] Moore CM, Baccinelli W, Sivokon O, Warnaar RSPW, Oppersma E (2023) ReSurfEMG. Doi: 10.5281/zenodo.6811553

[107] Betts JG, Desaix P, Johnson E (Edward W), et al (2022) Anatomy and physiology.

[108] LoMauro A, Aliverti A (2019). Respiratory muscle activation and action during voluntary cough in healthy humans. J Electromyogr Kinesiol.

[109] Mohammadi P, Akbari M, Sarrafzadeh J, Moradi Z (2014). Comparison of respiratory muscles activity and exercise capacity in patients with idiopathic scoliosis and healthy individuals. Physiother Theory Pract.

[110] Stewart H, Eisen A, Road J, Mezei M, Weber M (2001). Electromyography of respiratory muscles in amyotrophic lateral sclerosis. J Neurol Sci.

[111] Maarsingh EJW, Van Eykern LA, Sprikkelman AB, Hoekstra MO, Van Aalderen WMC (2000). Respiratory muscle activity measured with a noninvasive EMG technique: technical aspects and reproducibility. J Appl Physiol.

[112] Sekiguchi H, Tamaki Y, Kondo Y, Nakamura H, Hanashiro K, Yonemoto K, Moritani T, Kukita I (2018). Surface electromyographic evaluation of the neuromuscular activation of the inspiratory muscles during progressively increased inspiratory flow under inspiratory-resistive loading. Physiol Int.

[113] Hawkes EZ, Nowicky AV, McConnell AK (2007). Diaphragm and intercostal surface EMG and muscle performance after acute inspiratory muscle loading. Respir Physiol Neurobiol.

[114] Chiti L, Biondi G, Morelot-Panzini C, Raux M, Similowski T, Hug F (2008). Scalene muscle activity during progressive inspiratory loading under pressure support ventilation in normal humans. Respir Physiol Neurobiol.

[115] Segizbaeva MO, Aleksandrova NP (2014). Inspiratory muscle resistance to fatigue during exercise and simulated airway obstruction. Hum Physiol.

[116] Jolley CJ, Luo YM, Steier J, Rafferty GF, Polkey MI, Moxham J (2015). Neural respiratory drive and breathlessness in COPD. Eur Respir J.

[117] Luiso D, Villanueva JA, Belarte-Tornero LC, Fort A, Blázquez-Bermejo Z, Ruiz S, Farré R, Rigau J, Martí-Almor J, Farré N (2020). Surface respiratory electromyography and dyspnea in acute heart failure patients. PLoS One.

[118] Maarsingh EJW, Van Eykern LA, De Haan RJ, Griffioen RW, Hoekstra MO, Van Aalderen WMC (2002). Airflow limitation in asthmatic children assessed with a non-invasive EMG technique. Respir Physiol Neurobiol.

[119] Maarsingh EJW, van Eykern LA, Sprikkelman AB, van Aalderen WMC (2004). Histamine induced airway response in pre-school children assessed by a non-invasive EMG technique. Respir Med.

[120] Schaer CE, Wüthrich TU, Beltrami FG, Spengler CM (2019). Effects of sprint-interval and endurance respiratory muscle training regimens. Med Sci Sports Exerc.

[121] Sajjadi E, Seven YB, Ehrbar JG, Wymer JP, Mitchell GS, Smith BK (2022). Acute intermittent hypoxia and respiratory muscle recruitment in people with amyotrophic lateral sclerosis: a preliminary study. Exp Neurol.

[122] Fokkema DS, Maarsingh EJW, van Eykern LA, van Aalderen WMC (2006). Different breathing patterns in healthy and asthmatic children: responses to an arithmetic task. Respir Med.

[123] Hug F, Raux M, Morelot-Panzini C, Similowski T (2011). Surface EMG to assess and quantify upper airway dilators activity during non-invasive ventilation. Respir Physiol Neurobiol.

[124] Ni Y, Shi G, Yu Y, Hao J, Chen T, Song H (2015). Clinical characteristics of patients with chronic obstructive pulmonary disease with comorbid bronchiectasis: a systemic review and meta-analysis. Int J Chron Obstruct Pulmon Dis.

