# Supplementary material for: Analysis and applications of respiratory surface EMG: report of a round table meeting
Source: Crit Care. 2024 Jan 2;28:2. doi: 10.1186/s13054-023-04779-x (PMC10759550; doi:10.1186/s13054-023-04779-x)
Supplement: Supplementary file 1 — Additional file 1. AF1_Background_expert_group. A comprehensive overview of the background, clinical field and academic degree of the expert group [file 13054_2023_4779_MOESM1_ESM.docx]

# Background expert group

| **Name** | **Profession** | **Clinical Field** | **Academic degree*** | **Affiliation** |
| --- | --- | --- | --- | --- |
| A.H. Jonkman | Technical physician | Adult intensive care | PhD | Department of Intensive Care Medicine, Erasmus Medical Center, Rotterdam, The Netherlands, |
| R.S.P. Warnaar | Technical physician | Adult intensive care | MSc | Cardiovascular and Respiratory Physiology, TechMed Centre, University of Twente, Enschede, The Netherlands, |
| W. Baccinelli | Research software engineer |  | PhD | Netherlands eScience Center, Amsterdam, The Netherlands |
| N.M. Carbon | Anesthesiologist | Adult intensive care | MD | Department of Anesthesiology, Friedrich Alexander-Universität Erlangen-Nürnberg, Uniklinikum Erlangen, Erlangen, Germany |
| R.F. D’Cruz | Respiratory physician | Pulmonology, Home mechanical ventilation | MD, PhD | Lane Fox Clinical Respiratory Physiology Research Centre, Guy’s and St Thomas’ NHS Foundation Trust, London, UK |
| J. Doorduin | Technical physician | Clinical neurophysiology | PhD | Department of Neurology, Donders Institute for Brain, Cognition and Behaviour, Radboud University Medical Center, Nijmegen, The Netherlands |
| J.L.M. van Doorn | Biomedical scientist | Clinical neurophysiology | MSc | Department of Neurology, Donders Institute for Brain, Cognition and Behaviour, Radboud University Medical Center, Nijmegen, The Netherlands |
| J. Elshof | Technical physician | Pulmonology, Home mechanical ventilation | MSc | Department of Pulmonary Diseases/Home Mechanical Ventilation, University of Groningen, University Medical Center Groningen, Groningen, The Netherlands |
| L. Estrada-Petrocelli | Biomedical engineer |  | PhD | Universidad Latina de Panamá (ULATINA), Facultad de Ingeniería and Secretaría Nacional de Ciencia, Tecnología e Innovación (SENACYT) - Sistema Nacional de Investigación (SNI), Panama, Panama |
| J. Graßhoff | Computer scientist |  | PhD | Fraunhofer Research Institution for Individualized and Cell-Based Medical Engineering, Lübeck, Germany |
| L.M.A. Heunks | Pulmonologist-intensivist | Adult intensive care | MD, PhD | Department of intensive care, Radboud University Medical Center, Nijmegen, the Netherlands |
| A.A. Koopman | Technical physician | Pediatric intensive care | MSc | Division of Paediatric Critical Care Medicine, Department of Paediatrics, Beatrix Children's Hospital, University Medical Center Groningen, Groningen, The Netherlands |
| D. Langer | Physiotherapist | Adult intensive care, Pulmonary Rehabilitation | PT, PhD | KU Leuven, Department of Rehabilitation Sciences, Research Group for Rehabilitation in Internal Disorders, B-3000 – Leuven (Belgium) |
| C.M. Moore | Research software engineer | Radiology | MD | Netherlands eScience Center, Amsterdam, The Netherlands |
| J.M. Nunez Silveira | Respiratory therapist | Adult intensive care | MSc | Hospital Italiano de Buenos Aires, Unidad de Terapia Intensiva, Ciudad de Buenos Aires, Argentina |
| E. Petersen | Computer scientist |  | PhD | Technical University of Denmark (DTU), DTU Compute, Kgs. Lyngby, 2800 Denmark |
| D. Poddighe | Physiotherapist | Adult intensive care, Pulmonary Rehabilitation | PT, MSc | KU Leuven, Department of Rehabilitation Sciences, Research Group for Rehabilitation in Internal Disorders, B-3000 – Leuven (Belgium). |
| M. Ramsay | Pulmonologist | Pulmonology, Home mechanical ventilation | MD, PhD | Lane Fox Clinical Respiratory Physiology Research Centre, Guy’s and St Thomas’ NHS Foundation Trust, London, UK |
| A. Rodrigues | Physiotherapist | Adult intensive care, Pulmonary Rehabilitation | PT, PhD | Keenan Centre for Biomedical Research, Li Ka Shing Knowledge Institute, Unity Health Toronto, Toronto, Ontario, Canada |
| L.H. Roesthuis | Technical physician | Adult intensive care | PhD | Department of intensive care, Radboud University Medical Center, Nijmegen, the Netherlands |
| A. Rossel | Internal medicine specialist, intensivist | Adult intensive care | MD | Department of Acute Medicine, Geneva University Hospitals, Geneva, Switzerland |
| A. Torres | Biomedical engineer |  | PhD | Universitat Politècnica de Catalunya ·BarcelonaTech (UPC), Institut de Bioenginyeria de Catalunya (IBEC), Barcelona Institute of Science and Technology (BIST) and Biomedical Research Networking Centre in Bioengineering, Biomaterials and Nanomedicine (CIBER-BBN), Barcelona, Spain |
| M.L. Duiverman | Pulmonologist | Pulmonology, Home mechanical ventilation | MD, PhD | Department of Pulmonary Diseases/Home Mechanical Ventilation, University of Groningen, University Medical Center Groningen, Groningen, The Netherlands |
| E. Oppersma | Technical physician | Adult intensive care/Home mechanical ventilation | PhD | Cardiovascular and Respiratory Physiology, TechMed Centre, University of Twente, Enschede, The Netherlands, |

* MD = medical doctor, PT = physiotherapist, PhD = Doctor of Philosophy, MSc = Master of Science
