# Supplementary figures and images for: Analysis and applications of respiratory surface EMG: report of a round table meeting
Source: Crit Care. 2024 Jan 2;28:2. doi: 10.1186/s13054-023-04779-x (PMC10759550; doi:10.1186/s13054-023-04779-x)

## Comparison of envelopes

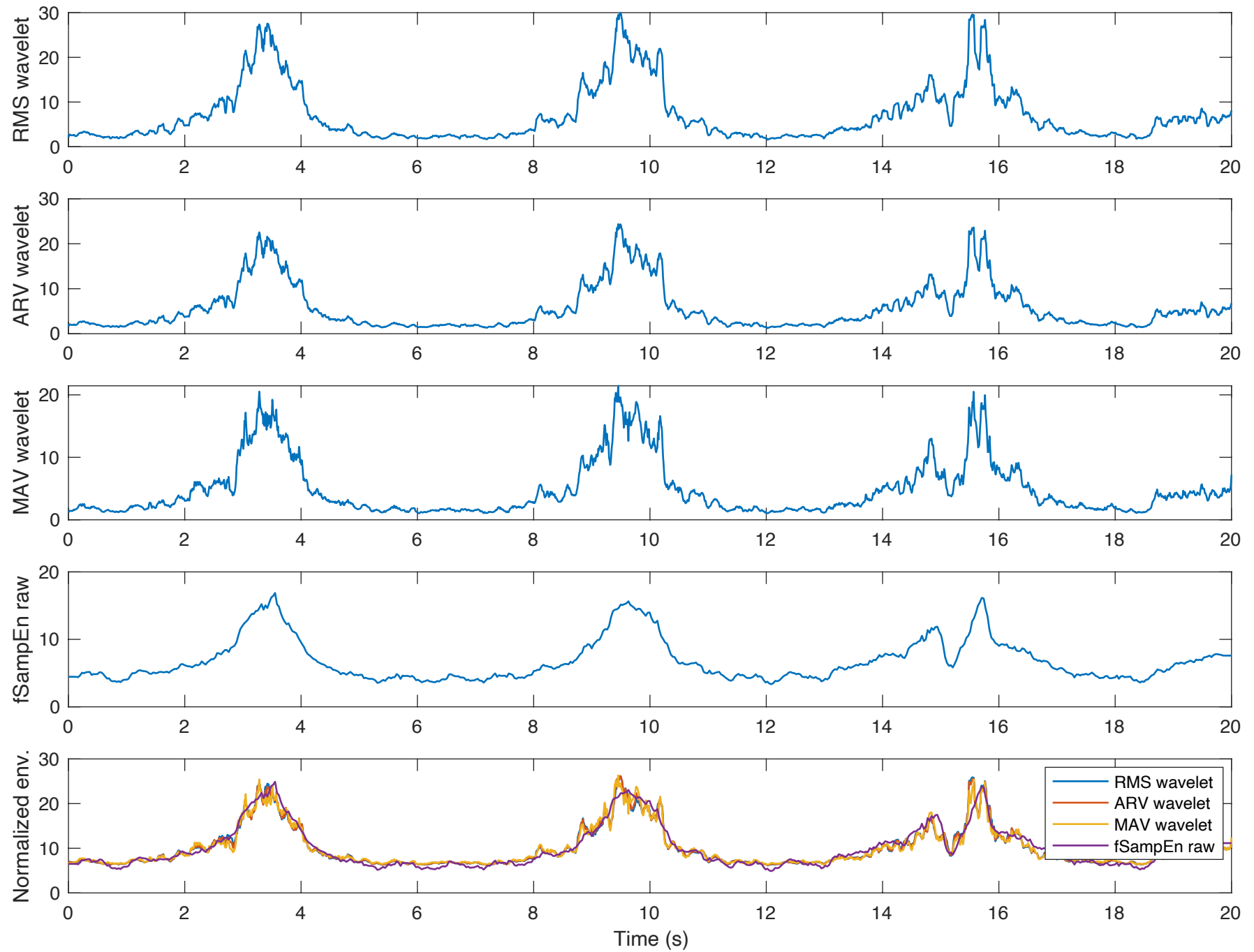

Supplement: Supplementary file 2 — Additional file 2. AF2_Comparison_envelope. A visual overview multiple methods for envelope calculation [file 13054_2023_4779_MOESM2_ESM.pdf]
